# Testing the Precedence Effect in the Median Plane Reveals Backward Spatial Masking of Sound

**DOI:** 10.1038/s41598-018-26834-2

**Published:** 2018-06-06

**Authors:** Rachel Ege, A. John van Opstal, Peter Bremen, Marc M. van Wanrooij

**Affiliations:** 1 Biophysics Department, Donders Institute for Brain, Cognition, and Behaviour, Radboud University, 6525 AJ Nijmegen, The Netherlands; 2 Department of Neuroscience, Erasmus Medical Center, P.O. Box 2040, 3000 CA Rotterdam, The Netherlands

## Abstract

Two synchronous sounds at different locations in the midsagittal plane induce a fused percept at a weighted-average position, with weights depending on relative sound intensities. In the horizontal plane, sound fusion (stereophony) disappears with a small onset asynchrony of 1–4 ms. The leading sound then fully determines the spatial percept (the precedence effect). Given that accurate localisation in the median plane requires an analysis of pinna-related spectral-shape cues, which takes ~25–30 ms of sound input to complete, we wondered at what time scale a precedence effect for elevation would manifest. Listeners localised the first of two sounds, with spatial disparities between 10–80 deg, and inter-stimulus delays between 0–320 ms. We demonstrate full fusion (averaging), and largest response variability, for onset asynchronies up to at least 40 ms for all spatial disparities. Weighted averaging persisted, and gradually decayed, for delays >160 ms, suggesting considerable backward masking. Moreover, response variability decreased with increasing delays. These results demonstrate that localisation undergoes substantial spatial blurring in the median plane by lagging sounds. Thus, the human auditory system, despite its high temporal resolution, is unable to spatially dissociate sounds in the midsagittal plane that co-occur within a time window of at least 160 ms.

## Introduction

**Figure 1:**
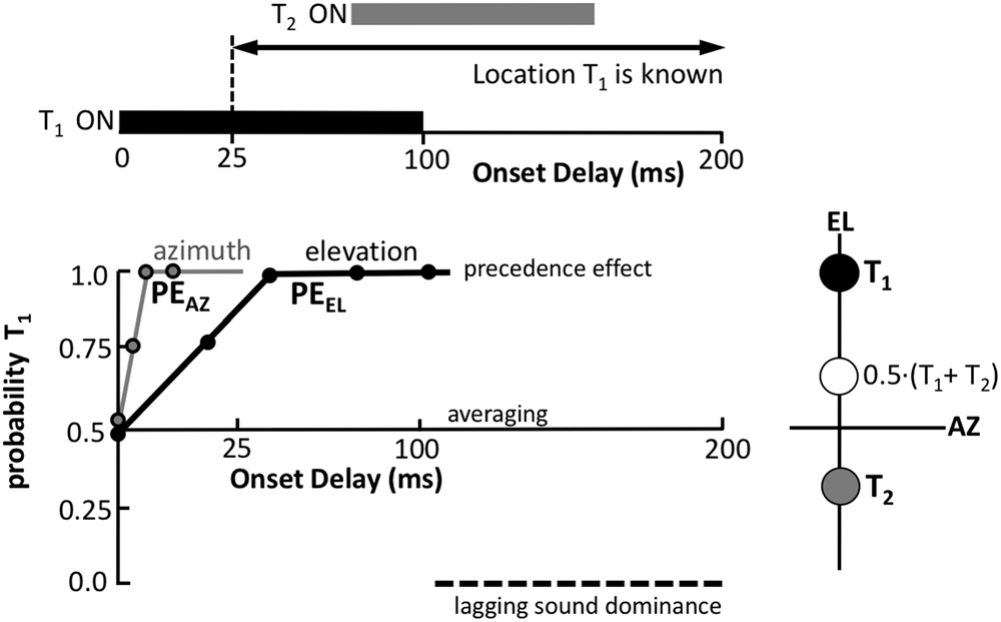
Rationale. Top: Leading-sound elevation (T_1_, 100 ms duration) is determined after ~30 ms. The lagging sound, T_2_, is delayed between 0–320 ms (here: 60 ms). Listeners localise T_1_. Bottom: Hypothetical precedence effect in elevation (PE_EL_) follows spectral-cue processing time, and weighted averaging of targets. After T_1_ offset, the lagging sound could potentially dominate the percept (dashed). In contrast, azimuth (grey line) is determined within a millisecond, and precedence rules after a few ms (PE_AZ_). Right: At 0 delay, targets average to a phantom percept at (T_1_ + T_2_)/2.

Synchronous presentation of two sounds from different locations is perceived as a fused (phantom) sound at the level-weighted average of the source locations. Weighted averaging has been demonstrated in the horizontal plane (azimuth, the stereophonic effect^1,2^), and in the midsagittal plane (elevation^3,4^). In the horizontal plane, already at onset asynchronies between 1–4 ms, the leading sound fully dominates localisation (Fig. 1^2,5–8^). This ‘precedence effect’ could provide a mechanism for localising sounds in reverberant environments^9^, as potential reflections are removed from the sound-location processing pathways.

Although asynchrony effects have been studied extensively in the horizontal plane (e.g.,^10–12^, in humans;^13^, in cats), little is known about their effects in the median plane. Extension to the latter is of interest, as the neural mechanisms underlying the extraction of the azimuth and elevation coordinates are fundamentally different, and initially processed by three independent brainstem pathways^14–16^. Whereas a sound’s azimuth angle is determined by interaural time (ITD) and level differences (ILD), its elevation estimate results from a pattern-recognition process of broadband spectral-shape information from the pinnae^17–21^.

An accurate elevation estimate is needed to disambiguate locations on the cone-of-confusion (the 2D surface on which the binaural differences are constant^2^). However, because the acoustic input results from a convolution of the sound-source (unknown to the system) and a direction-dependent pinna filter (also unknown), extraction of the veridical elevation angle from the sensory spectrum is an ill-posed problem^19^. To cope with this, the auditory system has to rely on additional assumptions regarding potential source spectra and sound locations, and its own pinna filters. As a result, elevation localisation requires several tens of milliseconds of acoustic input to complete^19,22,23^. In contrast, azimuth is accurately determined for sounds shorter than a millisecond (Fig. 1).

Some studies have reported a precedence effect in the midsagittal plane^7,24^. However, these studies included a limited range of locations, and the applied sound durations (<2 ms) may have been too brief for accurate localization^19^. Dizon and Litovsky^23^ included a larger range of locations, and sound durations up to 50 ms. They identified a weak precedence effect in the median plane, which, however, may also be described as weighted averaging, as there was no clear dominance of the leading sound.

We wondered how the fundamentally different localisation mechanism for elevation would affect the ability of the auditory system to segregate two sounds at different elevations, with temporal onset asynchronies. We reasoned that, at best, a precedence effect may emerge *after* the leading sound’s elevation had been determined, after ~30 ms (Fig. 1). We tested this prediction by asking listeners to localise the leading sound (the target), and ignore the lagging sound. To assess the influence of the latter on the localisation response, we employed a large range of inter-stimulus delays: (i) from synchronous presentation, up to 30 ms delay, (ii) delays with acoustic overlap of both sounds, but >30 ms, and (iii) full temporal segregation of the sounds (delays >100 ms).

## Results

### Single-Sound Localisation

**Figure 2:**
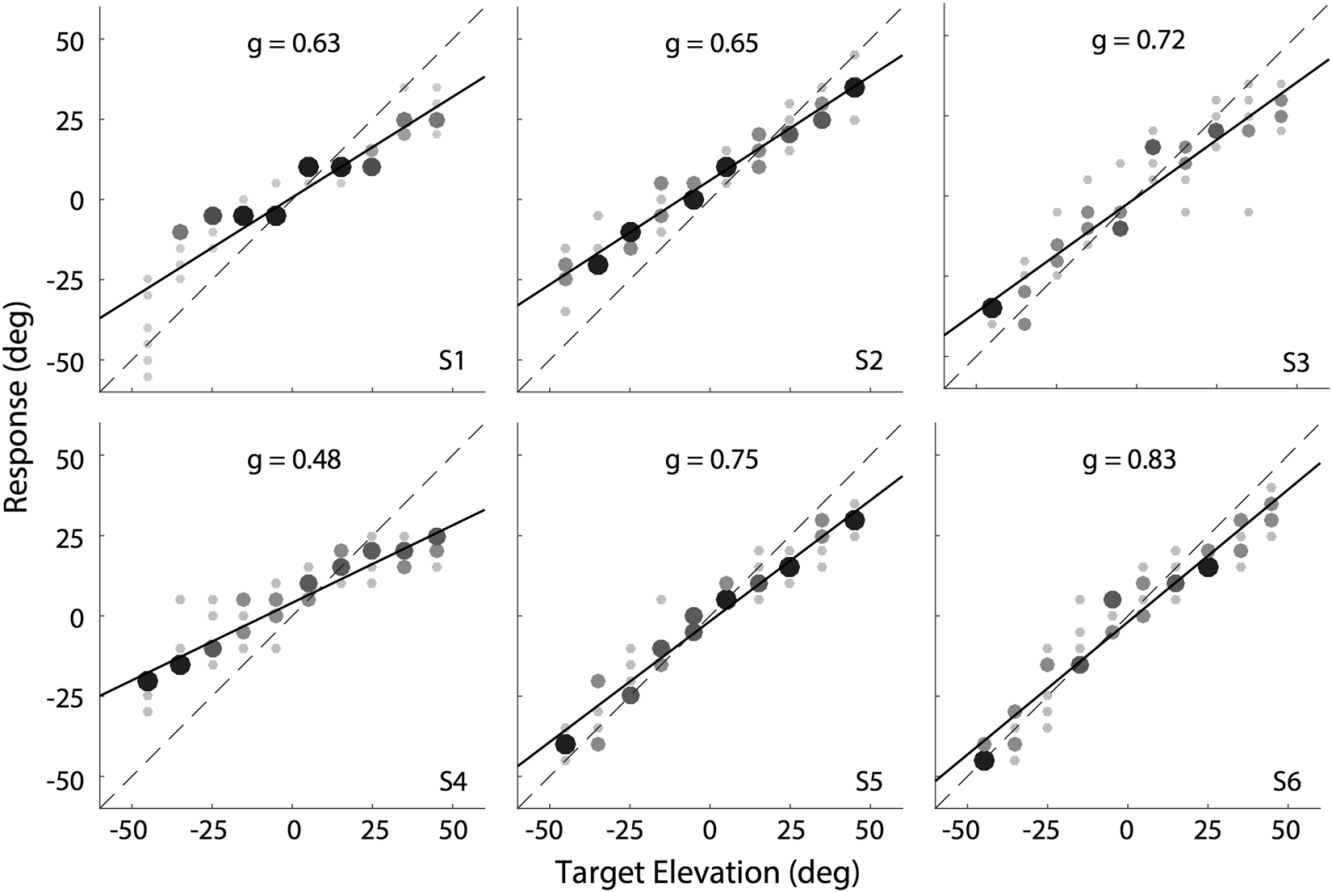
Single-speaker localisation performance. Individual stimulus-response relations for all subjects, pooled for single sounds (BZZ) and superimposed double-sounds (BZZ + GWN) at all delays. The data are displayed as bubble plots, in which the number of data points within each spatial bin is indicated by symbol size and grey code: the more/fewer responses in a bin, the larger/smaller and darker/lighter the symbol. Dashed diagonal indicates perfect behavior, the solid line corresponds to the optimal linear regression line through the data (Eqn. 1).

Figure 2 shows the single-speaker localisation results for all six participants. The single-sound trials (BZZ), and those in which the BZZ and GWN emanated from the same speaker, were all pooled, as we obtained no significant differences in response behavior for these trial types. All listeners showed a consistent linear stimulus-response relation, albeit that there were idiosyncratic differences in the absolute values of their localisation gains (range: 0.48–0.75). The biases were close to zero degrees for all participants. These results are in line with earlier reports^19,25^.

### Precedence vs. Weighted Averaging

In the double-sound trials, we presented the two sounds from different speakers with an onset delay between 0 and 320 ms (Methods).

**Figure 3:**
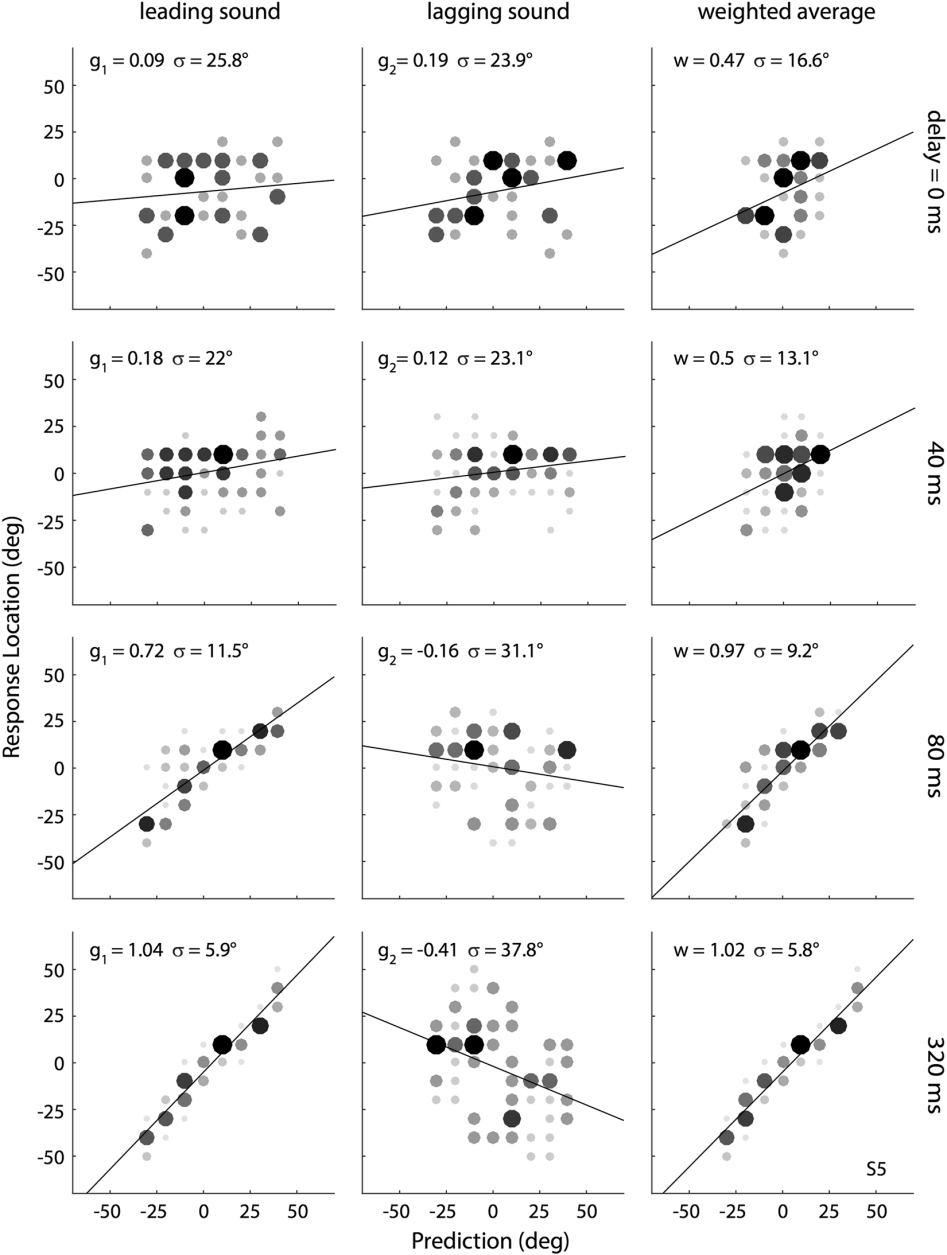
Stimulus-response relations to double-sounds. Each row shows the stimulus-response relation for a different onset delay between BZZ and GWN, emanating from different speakers. First and second column: results of the linear regressions (Eqn. 2), with responses plotted as function of the leading sound location, and lagging sound location, respectively. Note that for a delay of 0 ms, the sounds are synchronous, and the regressions refer to GWN (leading) and BZZ (lagging), respectively. Right-hand column: stimulus-response relation for the weighted-average model of Eqn. 3. Despite the considerable variability at the shorter delays, the weighted average model clearly outperforms the target-based partial regression fits. Data from S5.

To quantify the double-sound response behavior, we applied the two regression models (Eqns 2 and 3) to the data of each participant. Figure 3 shows the double-sound regression results for listener S5, for four selected onset delays, pooled for either sound stimulus as the leading source (132 trials per panel). The left-hand and center columns of this figure show the results of the linear regression analyses of Eqn. (2), applied to the leading sound (nr. 1, left) and lagging sound (nr. 2, center), respectively. The rows from top to bottom arrange the data across four selected onset delays (ΔT = 0, 40, 80, and 320 ms). At onset delays of 0 and 40 ms, the gains for the leading (g_1_) and lagging (g_2_) sounds were very low (close to zero), and did not appear to differ from each other. At a delay of 80 ms the weight for the leading sound increased to g_1_ = 0.72. At ΔT = 320 ms, the leading sound fully dominated the response (g_1_ = 1.04), and the listener’s performance became indistinguishable from single-speaker localisation (which corresponds to g_1_ = 1.0).

The right-hand column shows the results of the weighted-average model of Eqn. 3 to these same data sets. Although this model has only one free parameter, the weight w, it described the data consistently better than the single-target regression results at the shorter delays (0, 40, and 80 ms): it yielded a higher gain (weight), and had significantly less remaining variability (and thus a higher r^2^). Also for this model, at the longest delay of 320 ms, the weight of the leading sound (w = 1.02, with σ = 5.8 deg) was indistinguishable from 1.0, meaning that the responses were identical to the single-speaker responses.

The data in Fig. 3 indicate that at delays below ~80 ms the responses were neither directed at the leading sound source, nor at the lagging stimulus, but could be better described by weighted-averaging responses, with weights close to *w* = 0.5. Still, the precision of the weighted-average was not very high, as evidenced by the relatively large standard deviations (for ΔT = 0 ms: σ_AV_ = 16.6 deg; ΔT = 40 ms: σ_AV_ = 13.1 deg, and for ΔT = 80 ms: σ_AV_ = 9.2 deg), when compared to single-target response performance, achieved for ΔT = 320 ms, for which σ_AV_ = 5.8 deg.

### Backward masking

**Figure 4:**
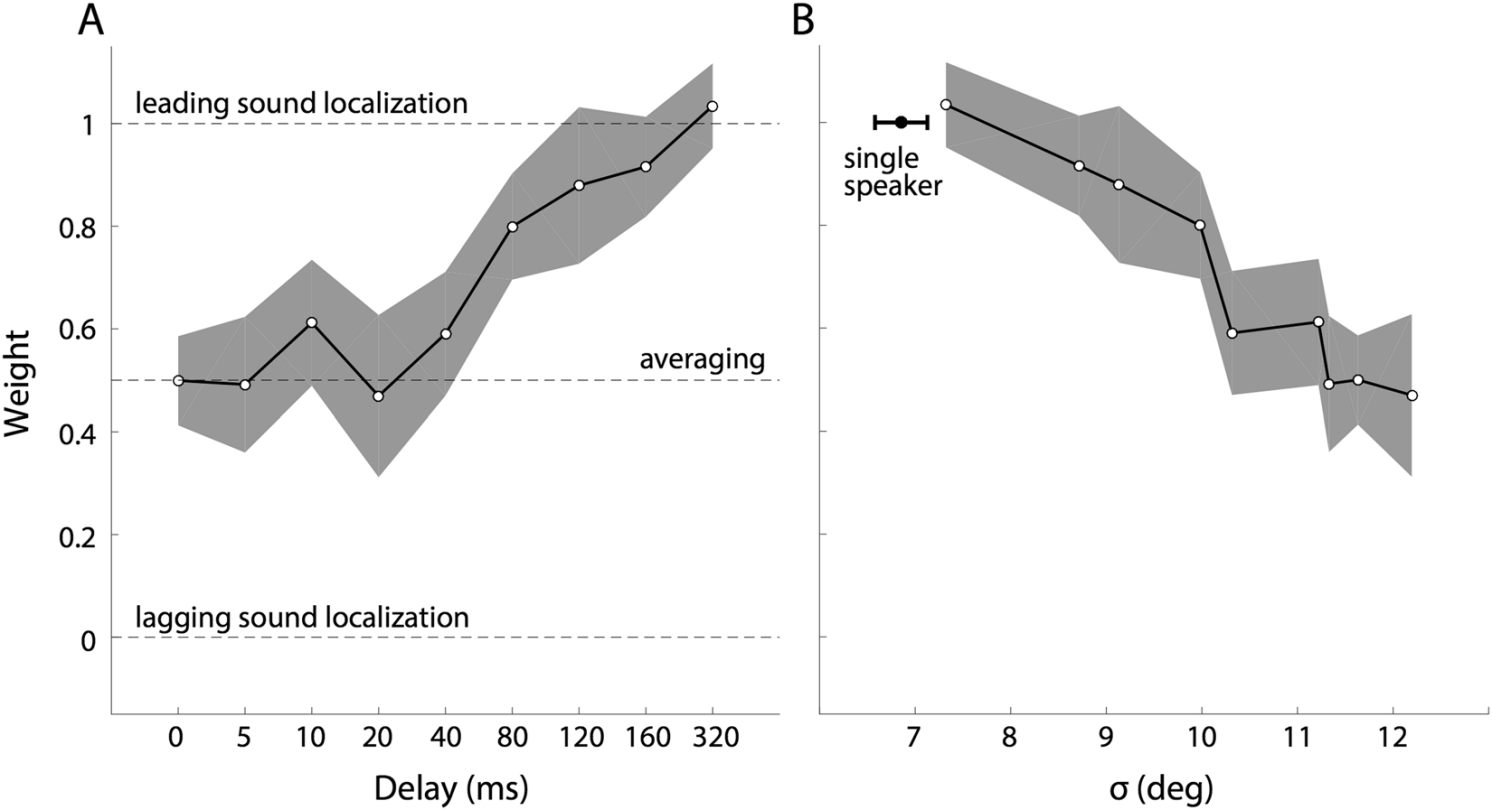
(**A**) Time-dependent weighted averaging. Results of the weighted-averaging model (Eqn. 3), averaged across subjects, for all onset asynchronies between 0 and 320 ms. Up to a delay of approximately 40 ms the weight remains close to 0.5, indicating full averaging. For delays >40 ms, the weight of the leading sound gradually increases. However, even at a delay of 160 ms, the lagging sound still influences response accuracy to the leading sound. Note that the delay is not represented on a linear scale. (**B**) Spatial blurring. Weight versus standard deviation of the response data around the model fit (Eqn. 3). The larger the weight, the smaller the standard deviation, hence the more precise the responses. Compare with Fig. 4, right-hand column. Filled dot: grand-averaged single-speaker localisation result with standard deviation.

Figure 4A shows how the weight of the leading sound, determined by Eqn. 3, varied as a function of the inter-stimulus delay, averaged across subjects. Up to a delay of ~40 ms, the responses are best described by the average response location, as the weights remain close to a value of 0.5. Note that the weight of the leading sound gradually increases with increasing delay. Yet, even at a delay of 160 ms, the leading-target weight is still smaller than 1.0 (w_AVG_ = 0.82), indicating a persisting influence of the lagging sound on the subject’s task performance.

The data in Fig. 3 (right-hand side) also suggest that the variability of the data around the model fit is considerable (>13 deg, for S5), especially at the shorter delays. This indicates that at delays <80 ms the weighted-averaging phantom source may not be perceived as spatially precise as a real physical sound source at that location, for which the standard deviation would be about 6 deg, or less. Figure 4B captures this aspect of the data for all participants, by plotting the relationship between the standard deviation and the weight for each delay. A consistent relation emerges between the variability in the double-sound responses, and the value of the leading-sound weight, which is indicative of a delay-dependent ‘spatial smearing’ of the perceived location. The shorter the delay, the larger the variability, and the closer the weight is to the average value of *w* = *0.5*. Conversely, the larger the onset delay, the better and more precise sound-localisation performance becomes (w_AVG_ ~1.0, and σ_AVG_ < 8 deg).

The data in Fig. 4 show that the auditory system is unable to dissociate sound sources in the median plane when they co-occur within a temporal window of up to ~160 ms. This poor localisation performance to double-sound stimulation is evidenced in two ways: weighted averaging, which leads to systematically wrong localisation responses (i.e., poor accuracy), and spatial blurring, leading to increased response variability at short asynchronies (i.e., poor precision). Yet, the auditory system has accurate and precise spatial knowledge of single sound sources within a few tens of ms (Fig. 2). The observed phenomenon thus seems to resemble backward spatial masking by the lagging sound on the spatial percept of a leading sound.

## Discussion

### Summary

Our results show that the leading source of two subsequent sounds, presented from different locations in the midsagittal plane, cannot be localised as accurately and precisely as a single source. For delays below 40 ms, subjects could not spatially segregate the sounds, as their responses showed full spatial averaging (*w* = 0.5). Overall, response behavior was best described by weighted averaging (Eqn. 3). Although both sound sources could have provided sufficient spectral information for adequate localisation, we did not observe bi-stable localisations, as head movements were not directed towards the lagging sound. Our results thus indicate a fundamentally different temporal sensitivity for localisation in the median plane, as compared to the horizontal plane (e.g.^8^).

### Precedence vs Backward masking

Accurate extraction of a sound’s elevation requires tens of milliseconds of broadband acoustic input^19,22,23,26^. In contrast, in the horizontal plane a localisation estimate is available within a millisecond^8^. Clearly, these differences originate in the underlying neural mechanisms^14–16^; while azimuth is determined by frequency-specific binaural difference comparisons, elevation requires spectral pattern evaluations across a broad range of frequencies between 3–15 kHz^21^. We reasoned that if 20–40 ms of acoustic input is required to determine elevation, it takes at least as long to assess whether the sound originated from a single or from multiple sources (Fig. 1). Indeed (Fig. 4A), up to about 40 ms, the auditory system is unable to differentiate sounds, resulting in the same averaged phantom percept as synchronous sounds of equal intensity^3,4^.

**Figure 5:**
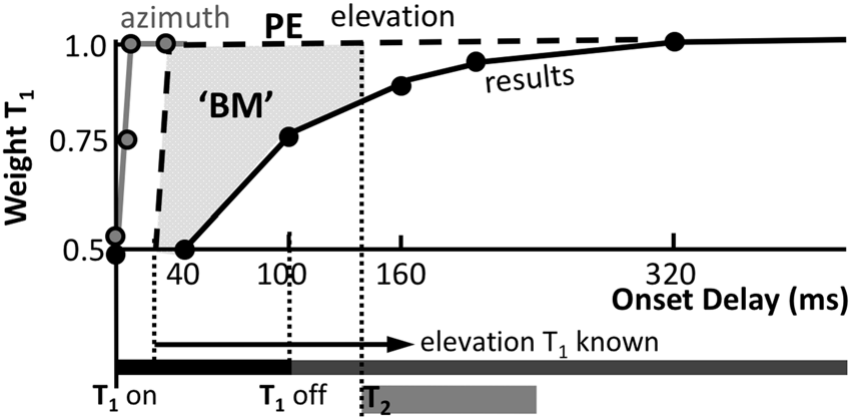
Processing of auditory localisation cues takes place at very different time scales for azimuth (grey) and elevation (black; cf. with Fig. 1). Although the location of the leading sound is available to the system after ~25–30 ms (black arrow, and dark-grey bar after T_1_ off), the lagging sound (here at ~140 ms) interferes with this process, even long after the leading sound’s offset. The grey patch between the alleged precedence effect (PE) for elevation, and the measured results indicates the strength of ‘backward masking’ (‘BM’).

Yet, we observed no precedence effect for elevation (Fig. 1), as beyond the 40 ms onset delay, the leading sound did not dominate localisation. Instead, responses were gradually directed more and more towards the leading sound, which, on average, took about 160 ms to complete. The sound durations in our experiments were 100 ms. In azimuth, such relatively long stimuli evoke strong precedence effects, also for time-overlapping sounds (e.g.^8,27,28^). This duration was more than sufficient to localise the leading sound (black bar) when presented in isolation (Fig. 2 ^19^;). Thus, the wide range of delays in our experiments (0–320 ms; grey bars) should have left the auditory system ample time to extract accurate spatial information of the leading sound (horizontal arrow in Figs 1 and 5). Yet, the lagging sound strongly interfered with the spatial percept of the leading source, even when it appeared long after spectral processing of the latter was complete. For example, at *∆T* = 160 ms, the acoustic input of the leading sound had disappeared for 60 ms. Its location would have been established ~120 ms earlier, as the auditory system had no prior information about a second sound in the trial. Indeed, without the latter, the leading sound would have been accurately localised. Yet, presentation of the distractor at this time point, still reduced the response gain for the leading sound by almost 15%, as *w* *~* *0.85*, as if, in retrospect, the auditory system re-evaluated its spatial estimate. As such, the observed phenomenon, highlighted in Fig. 5, seems to resemble a remarkably strong form of ‘*backward spatial masking*’^29,30^.

This persistent influence of a lagging sound on the perceived leading sound’s location has no equivalent in the horizontal plane (grey circles in Fig. 5). There, precedence dictates that a brief onset delay of a few ms suffices for full dominance of the leading sound^6^ (see^8,12^ for reviews). For synchronous sounds in the horizontal plane one observes, like in the median plane, level-weighted averaging^2^. The transition from pure averaging to full first wave-front dominance rapidly evolves for onset delays <1 ms.

### Comparison with cats

Tollin and Yin^13^ showed that cats perceive the precedence effect in azimuth, just like humans: up to a delay of ~0.4 ms, the cat perceives a weighted average location, which turns into full dominance of the leading sound for delays up to 10 ms. Beyond this delay (the echo threshold), the cat localises either sound. Unlike humans, however, cats display the same short-delay precedence phenomenon in elevation as in azimuth, albeit without averaging at extremely short delays. This result contrasts markedly with our findings (Figs 4 and 5).

A cat’s pinna-related spectral cues are differently organized than those of humans^17,20,31^. Whereas the elevation-specific spectral-shape information from the human pinna is encoded over a wide frequency bandwidth^21^, the major pinna cue in the cat is a narrow notch region that defines a unique iso-frequency contour in azimuth-elevation space^31^. Possibly, frequency-specific notch-detection in the cat’s early auditory system (presumably within the dorsal cochlear nucleus, e.g.^15^) might have similar delay sensitivity than the frequency-specific ITD or ILD pathways for azimuth. Moreover, the cat’s localization performance in elevation seems quite robust to very brief (<5 ms) sound bursts^13^. Although humans are capable of localizing brief (<10–20 ms) broadband sounds in elevation for levels below about 40 dB sensation level, their performance for brief sounds degrades at higher sound levels^19,22,23,32^.

### Neural Mechanisms

Clearly, this long-duration backward masking in the median plane (Figs 4 and 5) cannot be accounted for by purely (linear) acoustic interactions at the pinnae^3^. Cochlear nonlinearities, which would potentially smear the spectral representations of time-overlapping inputs^26,32^, cannot explain these effects either, as the cochlear excitation patterns from the leading sound will have died out already a few ms after its offset.

It is also difficult to understand how interactions within a spatial neural map could account for the vastly different behaviors for azimuth and elevation. If weighted-averaging of stimuli would be due to time-, intensity-, and space-dependent interactions within a topographic map, both coordinates would show the same results. Indeed, such omnidirectional effects have been reported for eye movements to visual double stimuli^33–35^. These have been explained as neural interactions of target representations within the gaze-motor map of the midbrain superior colliculus^36^. Based on our results, averaging in the auditory system seems to differ fundamentally from the mechanisms within the visuomotor system, rather indicating neural interactions within the tonotopic auditory pathways.

As argued in the Introduction, estimating elevation from the sensory input is an ill-posed problem, even for a single sound source. Thus, the auditory system should make a number of intrinsic assumptions (priors) about sound sources and pinna filters to cope with this problem^18,37^. For example, Hofman and Van Opstal^19^ showed that as long as source spectra do not resemble head-related transfer functions (HRTFs), cross-correlating the sensory spectrum with stored HRTFs will peak at the veridical elevation of the sound. Multiple sound sources will likely give rise to multiple peaks in a cross-correlation analysis, so that additional decision and selection mechanisms should infer the most likely cause (or causes) underlying the sensory spectrum (a process called causal inference; e.g.^38^).

To resolve locations on the cone of confusion, spectral-shape information from the convolved sound source and elevation-specific pinna filter is required to disambiguate potential sound directions. Sound locations are thus specified by unique triplets of ILD, ITD and (inferred) HRTF. The midsagittal plane is the only plane for which both ILDs and ITDs are exactly zero. Clearly, in a natural acoustic environment it is highly unlikely that multiple sources would lie exactly in this plane. Thus, if the auditory system is confronted with a sound field for which ILD and ITD are both zero, the most likely (inferred) cause would be a single sound source. Synchrony of sounds further corroborates such an assumption. If causal inference would indeed underlie the analysis of acoustic input, in the median plane the auditory system would be strongly biased towards a single source. Hence, the system insists on strong evidence for the presence of independent sources, e.g., a long inter-stimulus delay.

Our data further suggest that the auditory system continuously collects evidence regarding the origin of acoustic input and that such an ongoing evaluation even continues after the leading sound disappears. Possibly, the system regards multiple sound bursts, separated by brief time intervals, as caused by a single source. Examples of such sounds abound in natural environments, like in human speech. The auditory system pays a small price for this strategy, in that it mislocalises multiple sources when they are presented exactly in the median plane. Such mislocalisations may then show up as ‘backward spatial masking’ by the lagging sound. Considering the low likelihood of this particular acoustic condition in natural sound fields, this seems a relatively small price to pay.

## Materials and Methods

### Participants

We collected data from six adult participants (three female; age: 26–30 yrs.; mean = 27.8 yrs.). All listeners had normal or corrected-to-normal vision, and no hearing dysfunctions, which was tested with a standard audiogram, and a standard sound-localisation experiment to broadband Gaussian white noise (GWN) sound bursts of 50 ms duration in the frontal hemifield. One participant (S1) is the first author of this study; the other participants were kept naive about the purpose of the study.

Prior to the experiments participants gave their written informed consent. The experimental protocols were approved by the local ethics committee of the Radboud University, Faculty of Social Sciences, nr. ECSW2016-2208-41. All experiments were conducted in accordance with the relevant guidelines and regulations.

### Apparatus

During the experiment, the subject sat comfortably in a chair in the center of a completely dark, sound attenuated room (L × W × H = 3.5 × 3.0 × 3.0 m). The floor, ceiling and walls were covered with sound-absorbing black foam (50 mm thick with 30-mm pyramids; AX2250, Uxem b.v., Lelystad, The Netherlands), effectively eliminating echoes for frequencies exceeding 500 Hz^39^. The room had an ambient background noise level below 30 dBA (measured with an SLM 1352 P, ISO-TECH sound-level meter). The chair was positioned at the center of a spherical frame (radius 1.5 m), on which 125 small broad-range loudspeakers (SC5.9; Visaton GmbH, Haan, Germany) were mounted. These speakers were organized in a grid by separating them from the nearest speakers by an angle of approximately 15 degrees in both azimuth and elevation according to the double-pole coordinate system^40^. Along the cardinal axes speakers were separated by 5 deg. Head movements were recorded with the magnetic search-coil technique^41^. To this end, the participant wore a lightweight spectacle frame with a small coil attached to its nose bridge. Three orthogonal pairs of square coils (6 mm^2^ wires, 3 × 3 m) were attached to the room’s edges to generate the horizontal (80 kHz), vertical (60 kHz) and frontal (48 kHz) magnetic fields, respectively. The horizontal and vertical head-coil signals were amplified and demodulated (EM7; Remmel Labs, Katy, TX, USA), low-pass-filtered at 150 Hz (custom built, fourth-order Butterworth), digitized (RA16GA and RA16; Tucker-Davis Technology, Alachua, FL, USA) and stored on hard disk at 6000 Hz/channel. A custom-written Matlab program, running on a PC (HP EliteDesk, California, United States) controlled data recording and storage, stimulus generation, and online visualisation of the recorded data.

### Stimuli

Acoustic stimuli were digitally generated by Tucker-Davis System 3 hardware (Tucker-Davis Technology, Alachua, FL, USA), consisting of two real-time processors (RP2.1, 48,828.125 Hz sampling rate), two stereo amplifiers (SA-1), four programmable attenuators (PA-5), and eight multiplexers (PM-2).

We presented two distinguishable frozen broadband (0.5–20 kHz) sound types during the experiment: a GWN, and a buzzer (20 ms of Gaussian white noise, repeated five times, BZZ). Each sound had a 100-ms duration, was pre-generated and stored on disk, was presented at 50-dBA, and had 5-ms sine-squared onset, cosine-squared offset ramps. In double-sound trials, both sounds were presented with one out of 9 possible onset delays (ΔT = {0, 5, 10, 20, 40, 80, 120, 160, 320} ms), whereby the BZZ and GWN could either serve as target (leading), or distractor (lagging). In double-sound single-speaker trials, both sounds (including their delays) were presented by the same speaker (the presented sound was the sum of the GWN and BZZ). In single-sound control trials we only presented the BZZ as the target.

Visual stimuli consisted of green LEDs (wavelength 565 nm; Kingsbright Electronic Co., LTD., Taiwan) mounted at the center of each speaker (luminance 1.4 cd/m^2^), which served as independent visual fixation stimuli during the calibration experiment, or as a central fixation stimulus during the sound-localisation experiments.

### Calibration

To establish the off-line calibration that maps the raw coil signals onto known target locations, subjects pointed with their head towards 24 LED locations in the frontal hemifield (separated by approximately 30 deg in both azimuth and elevation), using a red laser, which was attached to the spectacle frame. A three-layer neural network, implemented in Matlab, was trained to carry out the required mapping of the raw initial and final head positions onto the (known) LED azimuth and elevation angles with a precision of 1.0 deg, or better. The weights of the network were subsequently used to map all head-movement voltages to degrees.

## Experimental Design and Statistical Analysis

### Paradigms

**Figure 6:**
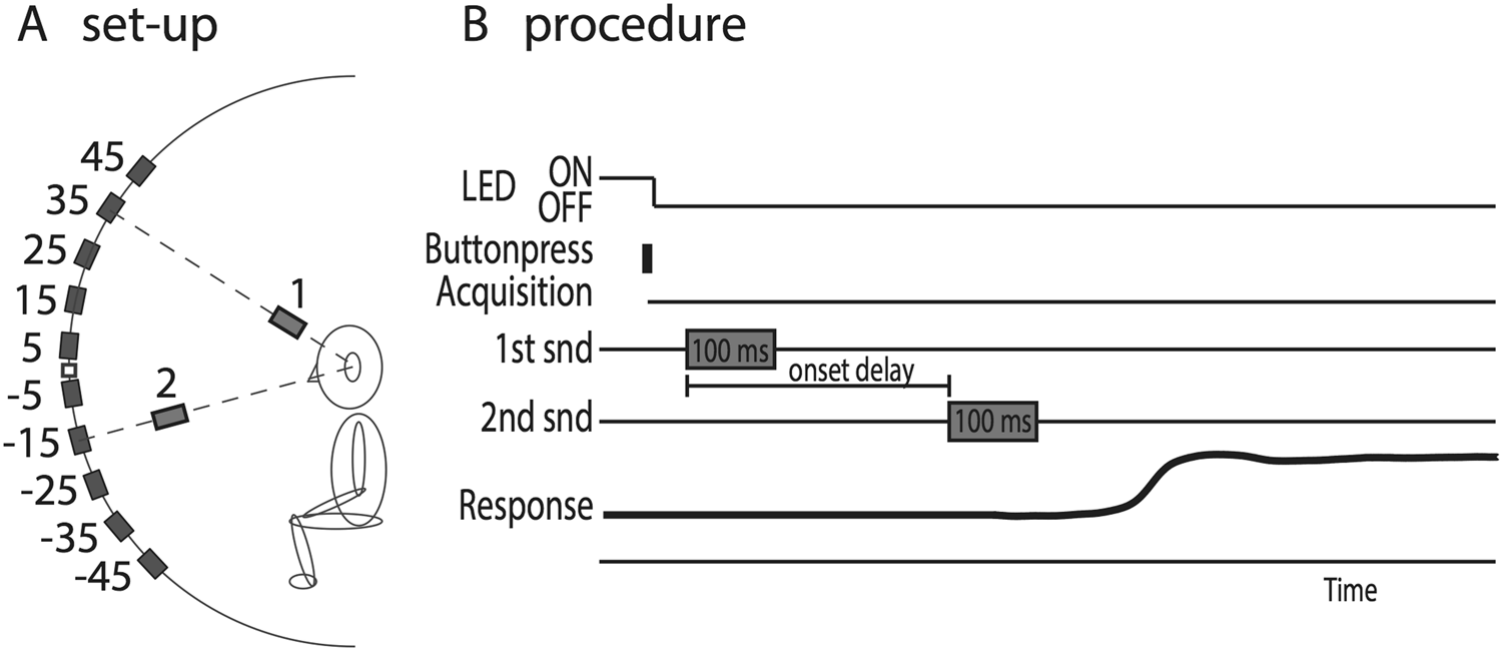
Experimental design of double-sound paradigm. (**A**) Speaker locations in the midsagittal plane ranged from −45 deg to +45 deg elevation, in steps of 5 deg, yielding 66 different double-speaker combinations. In the example, the leading sound (1) is presented at +35 deg, the lagging sound (2) at −15 deg (spatial disparity of 50 deg). (**B**) The listener initiated a trial by pressing a button after having aligned a head-mounted laser pointer with a straight-ahead fixation LED. The LED switched off 200 ms later. After a 200 ms gap, the leading sound turned on for 100 ms. The lagging sound followed with a varying delay. The subject had to make a rapid goal-directed head movement towards the perceived leading sound.

Participants were instructed to first align the head-fixed laser pointer with the central fixation LED. The fixation light was extinguished 200 ms after the participant pressed a button (Fig. 6B). After another 200 ms, the first sound was presented (either GWN, or BZZ), followed by a second, delayed sound (BZZ, or GWN, respectively). Sounds were presented by pseudorandom selection of two out of ten speaker locations in elevation ranging from −45 to +45 deg in 10 deg steps (Fig. 6A; the applied spatial disparities were 10, 20, 40, 50, 70 and 80 deg).

Participants were instructed to “point the head-fixed laser as fast and as accurately as possible towards the perceived location of the *first* sound source”. Data acquisition ended 1500 ms after the *first*-sound onset, upon which a new trial was initiated, after a brief inter-trial interval of between 0.5 and 1.5 s.

All participants underwent a short practice session of 25 randomly selected trials. The purpose of this training was to familiarize them with the open-loop experimental procedure, and their task during the experiment. No explicit feedback was provided about the accuracy of their responses. They were encouraged to produce brisk head-movement responses with fast reaction times, followed by a brief period of fixation at the perceived location.

Like in our earlier study, using a synchronous GWN and buzzer in the midsagittal plane^3^, subjects did not report having perceived any of the sounds as coming from the rear, which would have hampered the accuracy and reaction times of their head-movement responses (they were able to turn around in the setup, if needed). When asked, they described having had clear spatial percepts of all sounds. We therefore believe that the results reported here were not contaminated by potential front-back confusions.

The main experiment consisted of 1482 randomly interleaved trials [1122 two-speaker double-sound stimuli, plus 340 single-speaker double sounds, and 20 single-speaker single-sound locations], divided into four blocks of approximately equal length (~370 trials). Completion of each block took approximately 25 minutes. Participants completed one or two blocks per day, resulting in two to four sessions per participant.

### Analysis

All data analysis and visualisation were performed in Matlab. The raw head-position signals (voltages) were first low-pass filtered (cut-off frequency 75 Hz) and then calibrated to degrees for azimuth and elevation (see above). A custom-written Matlab program detected the head-movement onsets and offsets in all recorded trials, whenever the head velocity first exceeded 20 deg/s, or first fell below 20 deg/s after a detected onset, respectively. We took the end position of the first goal-directed movement after stimulus onset as a measure for sound-localisation performance. Each movement-detection marking was visually checked by the experimenter (without having explicit access to stimulus information), and adjusted when deemed necessary. In about 6% of the trials (single- and double-speaker conditions), a second head-movement response was present. This second response was not included as a true localisation response in the regression analyses discussed below.

### Statistics

The optimal linear fit of the stimulus-response relation for all pooled single-speaker responses (N = 360) was described by:

1 $${\varepsilon }_{R}=g\cdot {\varepsilon }_{T}+b\,$$

The slope (or gain), *g* (dimensionless), of the stimulus-response relation quantifies the sensitivity (resolution) of the audiomotor system to changes in target position; the offset, b (in deg), is a measure for the listener’s response bias. We fitted the parameters of Eqn. 1 by employing the least-squares error criterion. Perfect localisation performance yields a gain of 1 and a bias of 0 deg. The standard deviation of the responses around the regression line, and the coefficient of determination, r^2^, with r Pearson’s linear correlation coefficient between stimulus and response, quantify the precision of the stimulus-response relation. The accuracy of a response is determined by its absolute error, |ε_T_ − ε_R_|, with ε_T_ and ε_R_ target elevation and response elevation, respectively.

To quantify whether the leading sound fully dominated the localisation response (precedence), or whether the lagging sound affects the perceived location in a delay-dependent manner (weighted averaging), we employed two regression models for each delay separately (66 trials for ΔT = 0 ms, 132 trials for each of the nonzero delays).

First, to assess precedence, we obtained the contribution of the leading sound, ${\varepsilon }_{S1}$, to the subject’s response, ${\varepsilon }_{R}$, through linear regression:

2 $${\varepsilon }_{R}=g\cdot {g}_{1}\cdot {\varepsilon }_{S1}+b$$

with ${g}_{1}={g}_{1}({\rm{\Delta }}T)$ the delay-dependent gain for the first target location, and g and b the gain and bias obtained for the single-sound responses (Eqn. 1). A similar regression was performed on the lagging sound, ${\varepsilon }_{S2},\,$yielding ${g}_{2}({\rm{\Delta }}T)$, to quantify a potential dominance of the lagging sound (see Fig. 1). By incorporating the result of Eqn. 1, we accounted for the fact that the perceived single-sound location, as measured by the goal-directed head-movement, typically differs from the physical sound location, and between listeners, as g and b often differ from their ideal values of 1 and 0, respectively.

Second, in the weighted-averaging model we allowed for a contribution of the lagging sound, while constraining the gains for the leading and lagging sounds, as follows:

3 $${\varepsilon }_{R}=g\cdot (w\cdot {\varepsilon }_{S1}+(1-w)\cdot {\varepsilon }_{S2})+b$$

with *w* = *w*(*ΔT*) the weight of the leading sound (the target, *ε*_*S*1_), which was considered to be a function of the delay, *ΔT*, and served as the only free parameter in this regression. Again, the single-target gain, g, and bias, b, of Eqn. 1 were included to calculate the perceived location of a single target at the weighted-average position, and to allow for a direct comparison with the single-speaker responses, and between subjects. If *w* = *1*, the response is directed toward the perceived first target location, and responses are indistinguishable from the single-target responses to that target. On the other hand, if *w* = *0*, the response is directed to the perceived location of the lagging distractor, and when *w* = *0.5*, responses are directed to the perceived midpoint between the two stimulus locations (averaging).

Statistical significance for the difference between the regression models (note that Eqn. 2 and Eqn. 3 both have only one free parameter) was determined on the basis of their coefficient of determination (*r*^*2*^).

### Data availability

The data sets analysed for the current study are available from the corresponding authors on reasonable request.
